# Supplementary material for: Poor outcomes among critically ill HIV-positive patients at hospital discharge and post-discharge in Guinea, Conakry: A retrospective cohort study
Source: PLoS One. 2023 Mar 13;18(3):e0281425. doi: 10.1371/journal.pone.0281425 (PMC10010544; doi:10.1371/journal.pone.0281425)
Supplement: Attachment — Submitted filename: Rebuttal letter.docx [file pone.0281425.s002.docx]

Rebuttal letter

We thank you for the opportunity to resubmit this article and hope you find the following responses to the peer review adequate.

**The following journal requirements were brought to our attention**:

1. *Please ensure that your manuscript meets PLOS ONE's style requirements, including those for file naming.*
2. *You indicated that ethical approval was not necessary for your study. We understand that the framework for ethical oversight requirements for studies of this type may differ depending on the setting and we would appreciate some further clarification regarding your research. Could you please provide confirmation from your institutional review board or research ethics committee (e.g., in the form of a letter or email correspondence) that ethics review was not necessary for this study? Please include a copy of the correspondence as an "Other" file.*
3. *Additionally, in the ethics statement in the manuscript and in the online submission form, please provide additional information about the patient records used in your retrospective study, including:*

*a) whether all data were fully anonymized before you accessed them;*

*b) the date range (month and year) during which patients' medical records were accessed.*

4. *To comply with PLOS ONE submission guidelines, in your Methods section, please provide additional information regarding your statistical analyses.*
5. *We note that you have indicated that data from this study are available upon request. PLOS only allows data to be available upon request if there are legal or ethical restrictions on sharing data publicly*
6. *In your revised cover letter, please address the following prompts:*

*a) If there are ethical or legal restrictions on sharing a de-identified data set, please explain them in detail (e.g., data contain potentially identifying or sensitive patient information) and who has imposed them (e.g., an ethics committee). Please also provide contact information for a data access committee, ethics committee, or other institutional body to which data requests may be sent.*

*b) If there are no restrictions, please upload the minimal anonymized data set necessary to replicate your study findings as either Supporting Information files or to a stable, public repository and provide us with the relevant URLs, DOIs, or accession numbers*

**Rebuttal to journal suggestions**

First there were several issues raised by the journal editors concerning Plos One’s style requirements which we addressed in compliance with the journals rules and regulations. Further we added the date range during which patients records were accessed for the study in the “data sources“ subsection of the methods part of the paper. We assured public availability of our data by uploading an anonymised dataset this study is based on (and commented on this in a revised cover letter). Also, additional information on statistical analysis was added, e.g. which software was used as well as researchers approach to missing data.

**Reviewer one had several remarks concerning different parts of our paper:**

1*. The authors should clearly describe the statement of problem and significance of the study in last paragraph of the introductory part.
2. Write abbreviations in full when it first appears (E.g. MSF).
Methods
1. Under this section, the authors need to include a number of sub topics which includes, sample size determination and sampling technique, data collection procedure and quality assurance, patient follow-up and outcome measurement and outcome validation
2. Line 57-“Data for this study was…” Data is plural. So, use were instead of was.
3. Line 63- Could the authors explain how they managed missing data?
4. Line 70-71- I suggest the Outcome definitions to put it under “outcome validation” separately.
5. The authors should provide the reference number for Ethical Committee Approval.
Results
1. Table: 1 is not easy to reader. Consider revising it. Revise also the topic,” Clinical characteristics and in-hospital outcomes..” since the table includes socio-demographic data.
2. Line 160-consider revising the sentence.
3. The authors should include the combined antiretroviral therapy (cART) regimens of the patients in a Table.
4. It was of interest if the authors could include the risk factors (predictors) of in-hospital mortality. This would help in identifying and tackling those risk factors. It is also mentioned under limitation as well.
5. Consider defining terminologies like malnutrition, and etc.
6. Could the authors determine the leading cause of death in the cohort?
Discussion
Your discussion is nice. Could you consider comparing the in-hospital death of AIDS patients in your study with those conducted in Eastern Africa (E.g. Negera GZ, [https://doi.org/10.1371/journal.pone.0226683](https://doi.org/10.1371/journal.pone.0226683)).*

**Rebuttal to reviewer one**

**Introduction**

Following the reviewers suggestion, we added a clear problem statement as well as a description of the scope and significance of our study. The reviewer also pointed out formal errors with abbreviations which we corrected.

**Methods**

We could not comply with the reviewers suggestion to include sample size calculations since this was a merely descriptive study of a retrospective cohort during the time period that the first authors were working in the project. Also, we continued to use data as a singular contrary to the reviewers suggestion since we prefer to use the word the way it is most often used in common parlance. Furthermore, we opted to not add a paragraph titled outcome validation as suggested, but we did add information to describe how LTFU has been validated.

We followed the reviewers advice and added additional information on how we dealt with missing data both in the methods section on statistical analysis as well as the outcome tables themselves. Also, the reference number for the approval of the national ethics committee was added to the text under the ethics section, plus further description of the process of approval from MSF ethics boards.

**Results**

Reviewer one voiced concerns regarding the title and readability of the tables. In order to address his remarks, we made several changes to our tables. For example, we removed sections of content from table 1 and 2 in order to facilitate readability. Also, we changed the tables header from “clinical characteristics...” to “clinical and demographic characteristics......“. Also the sentence in line 160 has been changed according to the reviewers suggestion.

Concerning the reviewers’ suggestion to put more information on patients ‘ART regiments in the table, we opted against augmenting the table in order to not complicated them further. We did however include an additional sentence on common 1st and 2nd line ART treatments in the introduction.

Furthermore, the reviewer suggested including a regression on mortality in the results. Since multivariate analyses of both hospital and follow-up outcomes did not reveal useful information beyond known associations of advanced disease (CD4 count) with mortality we opted against including these analyses in the paper and addressed this in the limitations section of the study.

The reviewer suggested we define clinical terms such as malnutrition and anaemia. We followed this suggestion and defined both terms in the results section of the paper as well as in table 3. Furthermore, we augmented the morbidity section to “morbidity and mortality and provided additional information on the leading cause of death during hospitalisation (Tuberculosis).

**Discussion**

The reviewer suggested additional references both on in-hospital mortality of HIV patients in other settings in sub-Saharan settings and to discuss further the influence of psychosocial factors on HIV care. We found the suggestions to be both instructive and pertinent and therefore included the suggested references and amended the discussion on psycho-social factors in our paper. Due to issues with the referencing software, being unable to cope when used in conjunction with track changes please note that the additional references were not added with “track changes” enabled but marked blue afterwards in the “track changes” version of the document. Such, they should still be easily identifiable.

**Reviewer two had several remarks concerning different parts of our paper**:

*Poor outcomes among critically ill HIV-positive patients at hospital discharge and post discharge in Guinea, Conakry: A retrospective cohort study” is a very informative and interesting paper that needs to fill some gaps before being published, in my opinion
The main gap is in the discussion of results, when the authors realized that the key point is the model of care which is clearly inadequate to the real world in Guinea. For example the high LTFU could be due simply to the lack of money for transport: however this information is not reported by the study as well as others and this should be reported as a limitation (which has been done only partially when the authors state “.. Finally, the actual outcome of those LTFU remains 271 uncertain due to the lack of active follow up.. “).
There is large number of papers who discuss the relation between social support joined with access to the standard of HAART care. I encourage the author to go in deep with this issue that that seems to be the key to improve HIV care in LRS
Other major gaps are related to the main one: the relevance of doling viral load on a routine basis to address the gap in timely switching to second line for example: is this a matter of the model of care or just the lack of reagents and laboratory able to perform it? To my knowledge of guinea (and especially Conakry) the laboratory should have enough potential capacity of performing Viral loads. However this should be discussed*

*Minor observations
Tables are some time so rich to became difficult to be read. The authors should consider the possibility of shifting part of data in a supplementary section in order to facilitate the readers in getting the key information
Please pay attention to percentage that in several cases do not round up to 100 (table 1 column “total” Row “VL cat copies/ml…..) for example
Some statements in the text are too much detailed, maybe
“..One case who initially was recorded as a case of chronic diarrhoea was diagnosed
with CM upon 2nd 175 hospitalization, while two TB cases were diagnosed with Toxoplasmosis
during their 2nd 176 hospitalization. Table 3 and 4 show that rates of death and attrition during and
177 after hospitalization are similar for all OIs. One exception is that patients with signs of severe
178 acute kidney injury had worse outcomes than patient with other conditions with 23 (57%)
179 p=0.002 dying during hospitalization, and 7 (50%) p=0.650 being LTFU or dead during the 6
180 months of follow up.”

Generally speaking it is best to give some less details to allow a better understanding
When you discuss the viability of ARV first line (NNRTI) please report the WHO guidelines to make understandable to the reader whether this is in agreement or not with the mainstream on this issue.*

**Rebuttal to reviewer two**

Reviewer two pointed out that our article was ambiguous as to whether viral load monitoring was readily available for patients in the study setting or not. We thank reviewer two for his astute observation and have amended the discussion clarifying that viral load monitoring was accessible for patients and clinicians, and we indicate that issues to do with access and use are therefore more complex and would benefit from further research.

Several other suggestions that reviewer two made such as further exploration of the influence of psychosocial factors in the discussion, and simplification of tables were in accordance with reviewer one, and we have made improvements to the tables and discussion as requested.

Reviewer two pointed out that in the tables, column percentages sometimes did not add up to 100%. After checking carefully, we are confident that this issue is solely due to our practice of rounding to zero decimals and not indicative of any systematic error.

Related to our results section, the reviewer suggested we simplify some of the description of complicated cases in order to facilitate understanding. While we share the reviewers concern, we feel that some of the more detailed descriptions convey important information and can be helpful in making the clinical reality of HIV care in this setting tangible for the reader.

Last, the reviewer suggests we add additional information on the WHO guidelines to our discussion on the viability of NNRTI based 1st line ART treatment. After reviewing the text, we found the existing references to the current WHO guidelines in both the introduction and discussion to be sufficient to give adequate context on current guidelines and practices. Further information was given to explain the regimens given to patients in this setting.
