# Supplementary material for: Poor outcomes among critically ill HIV-positive patients at hospital discharge and post-discharge in Guinea, Conakry: A retrospective cohort study
Source: PLoS One. 2023 Mar 13;18(3):e0281425. doi: 10.1371/journal.pone.0281425 (PMC10010544; doi:10.1371/journal.pone.0281425)
Supplement: Attachment — Submitted filename: Rebuttal letter to reviewers.docx [file pone.0281425.s003.docx]

Rebuttal letter to reviewers’ comments from 16.5.2022

We thank you for the opportunity to resubmit this article and hope you find the following responses to the peer review adequate:

Reviewer 2 asked whether it was possible to list the health units where the patients were followed up after discharge

Answer: We included a sentence in the methods section mentioning the name and geographic distribution of the community health centers that the study hospital received referrals from

Reviewer 2 asked whether it was possible to include reason for admission.

Answer: reasons for admission were not specified in the database that our study was based on. We did however add a sentence in the methods section describing admission criteria to USFR in general.

Reviewer 2 asked why malnutrition was defined with MUAC and not with BMI and wether BMI data was available. Also he suggested moving the definition of malnutrition in the methods section and not in the results. Further the reviewer asked why in this study we focused on the outcmes of hospitalised patients only and argued that this would make it difficult to generalize findings from our study to other populations and settings

Answer: we added a sentence in the methods section explaining why we chose MUAC over BMI in defining severe malnutrition. The definition of severe malnutrition was moved from the results to the methods section. In regards to our focus on hospitalized patients, we feel that patients with advanced HIV who require hospitalization are a vulnerable sub group of PLHIV who contribute overproportionally to HIV related mortality in high and income settings alike. While our findings might therefore not be generalized to other HIV populations, they might very well expand the knowledgebase on outcomes of critically ill patients with advanced HIV infection.
