# Supplementary material for: Poor outcomes among critically ill HIV-positive patients at hospital discharge and post-discharge in Guinea, Conakry: A retrospective cohort study
Source: PLoS One. 2023 Mar 13;18(3):e0281425. doi: 10.1371/journal.pone.0281425 (PMC10010544; doi:10.1371/journal.pone.0281425)
Supplement: Attachment — Submitted filename: rebuttal 220922.docx [file pone.0281425.s004.docx]

Rebuttal letter to remarks by reviewer 2 made on 2.9.2022

Reviewer 2 found the results section of the paper needing of improvement:

First reviewer 2 suggested changing table 1 and 2 so they would show both dead/LTFU as well as patients still alive. We appreciate the comment and changed both table 1 and 2 to the suggestion of reviewer 2. Please note however that as a consequence we struggled to fit the tables on A4 portrait page format. Please feel free to change the formatting accordingly to comply with journal requirements before publishing.

Second, reviewer 2 found the narrative description of the section „baseline demographic and clinical characteristics“ unclear. We appreciate his comments and improved the wording, including more information on the two cohorts that were included in the analysis. For details, we would like to referrreviewers to the uploaded document with track changes.
